# Robot-assisted percutaneous balloon compression for trigeminal neuralgia- preliminary experiences

**DOI:** 10.1186/s12883-023-03199-2

**Published:** 2023-04-22

**Authors:** Ning Li, Tao Sun, Bin Hu, Kun Zhao, Changming Zhang, Jinlong Liu, Chao Yang

**Affiliations:** 1 Department of Neurosurgery, First Affiliated Hospital of Sun Yat-Sen University, No 58th, Zhongshan Er Road, Yuexiu District, Guangzhou, 510080 China; 2 Department of Neurosurgery, Guangxi Hospital Division of The First Affiliated Hospital, Sun Yat-sen University, No 3rd Fozi Ling Road, Qingxiu District, Nanning, 530022 China

**Keywords:** Robot-assisted, Three-dimensional structured light registration, Percutaneous balloon compression, Foramen ovale, Trigeminal neuralgia

## Abstract

**Objectives:**

This study aims to discuss the availability of robot-assisted percutaneous balloon compression (PBC) for trigeminal neuralgia (TN) and share our preliminary experiences.

**Methods:**

Patients with TN who underwent robot-assisted PBC from June to September 2022 were enrolled. We designed a fixing plug for robot-assisted PBC, three-dimensional structured light registration was used, puncture trajectory was the line connects the medial third of inner and outer aperture of foramen ovale. Numerical Rating Scale (NRS), Barrow Neurological Institute (BNI) pain and numbness intensity score were used to evaluate the facial pain and numbness.

**Results:**

Eventually, nine patients were enrolled, the structured light registrations were successfully finished in all patients with a mean registration error of 0.68 mm. All the punctures of foramen ovales were successfully done one-time. Of note, the balloons were all got pear-shaped followed by 150 to 180 s compression. Though, postoperatively, all the patients complained of facial numbness and four patients suffered from transient masseter weakness, all patients got fully or mostly pain relief. It should be noted that is the numbness and weakness gradually relieved during follow-up.

**Conclusion:**

Three-dimensional structured light registration and robot assisted PBC is an effective choice for patients with TN. Extension line between the medial third of the inner and outer aperture of foramen ovale might be a safe and effective puncture trajectory to this procedure.

## Introduction

Trigeminal neuralgia (TN) as a paroxysmal severe pain syndrome restricted in the distributions of trigeminal nerve, badly interferes the quality of life of the patients, which mainly impacts the second and third branches of trigeminal nerve [1, 2]. According to the mechanism of TN and the morphological changes of the nerve, TN could be divided into classical, idiopathic and secondary TN [2]. Presently, oral medication is the first choice for TN [1]. As a minimal invasive method, percutaneous balloon compression (PBC) is also a practical option for TN [3]. Although the procedure can be easily done by experienced doctors, it requires a long learning cycle[4]. What’s more, this procedure could result in some severe complications, which are usually related to foramen ovale puncture [5–7]. For this, many devices and techniques have been used for it [8]. In current study, we aims to discuss the feasibility and availability of robot-assisted PBC and share our experiences toward it.

## Patients and methods

### Patients

We enrolled the patients with TN who underwent robot-assisted PBC in our department from June to September 2022. All patients signed informed consents, and this study was approved by the ethics committee of our hospital. The basic characteristics the patients were recorded. Preoperatively, the patients were enrolled for robot-assisted PBC based on following criteria.

Inclusion criteria:

1) TN was definitely diagnosed.

2) Regular oral medications were unsatisfactory.

3) Patients couldn’t tolerate oral medications or craniotomy.

4) Patients refused microvascular decompression (MVD).

Exclusion criteria:

1) Patients with local broken or infected skin.

2) Regular oral medication less than half a year.

3) Patients with severe comorbidity and can’t tolerate PBC.

4) Patients with significant coagulation dysfunction.

### Materials

**Fig. 1:**
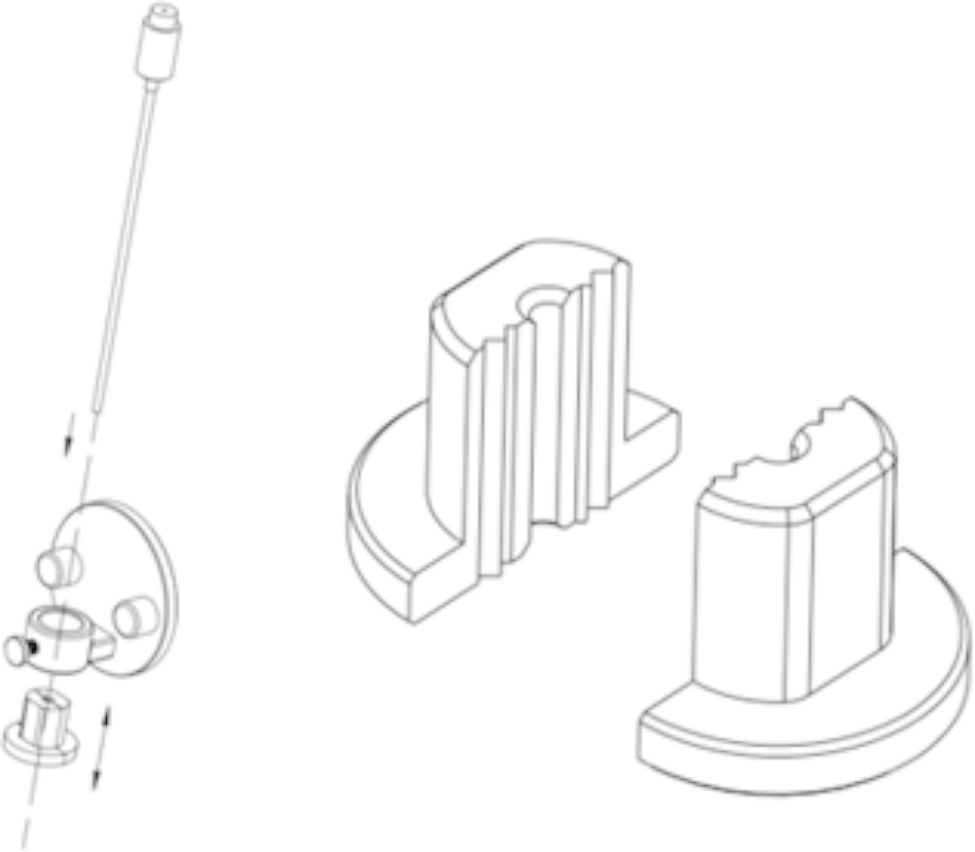
Schematic of the two-piece set of fixing plugs for robot-assisted PBC

**Fig. 2:**
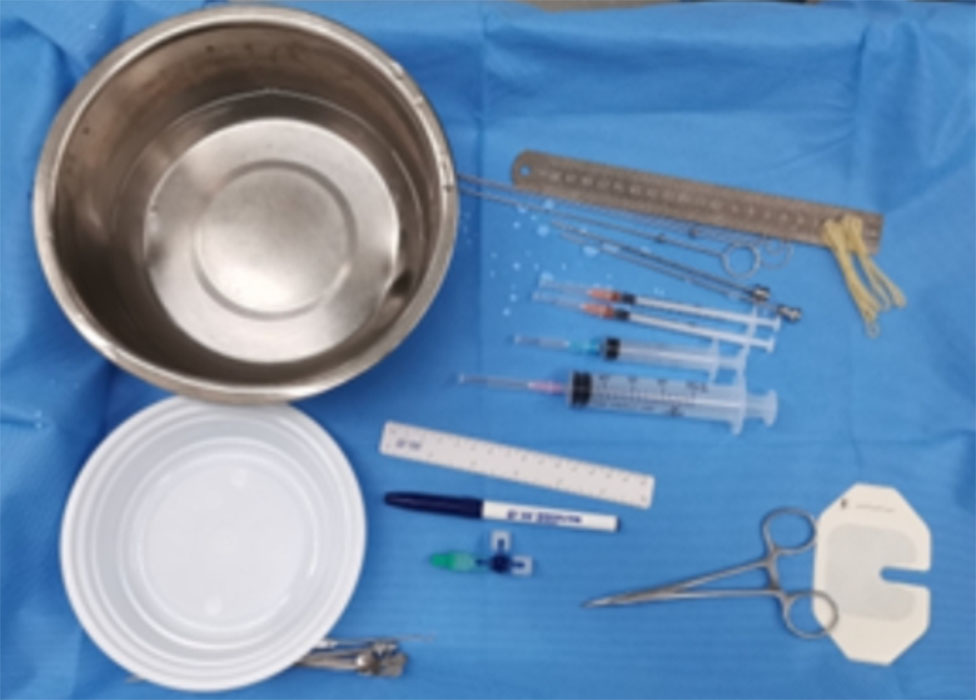
Other instruments of the procedure

We designed a fixing plug for three-dimensional structured light registration robot-assisted PBC, which could be assembled and firmly fixed on the robotic arm (Fig. 1). C-arm set x-ray machine (Ziehm, Germany), Sinovation neurosurgical robot (Sinovation, Beijing, China), one-time neurosurgical balloon catheter (Cerebral Corridor Creator, Shenzhen, China), DORO head frame and other materials (Fig. 2).

### Puncture trajectory designations

**Fig. 3:**
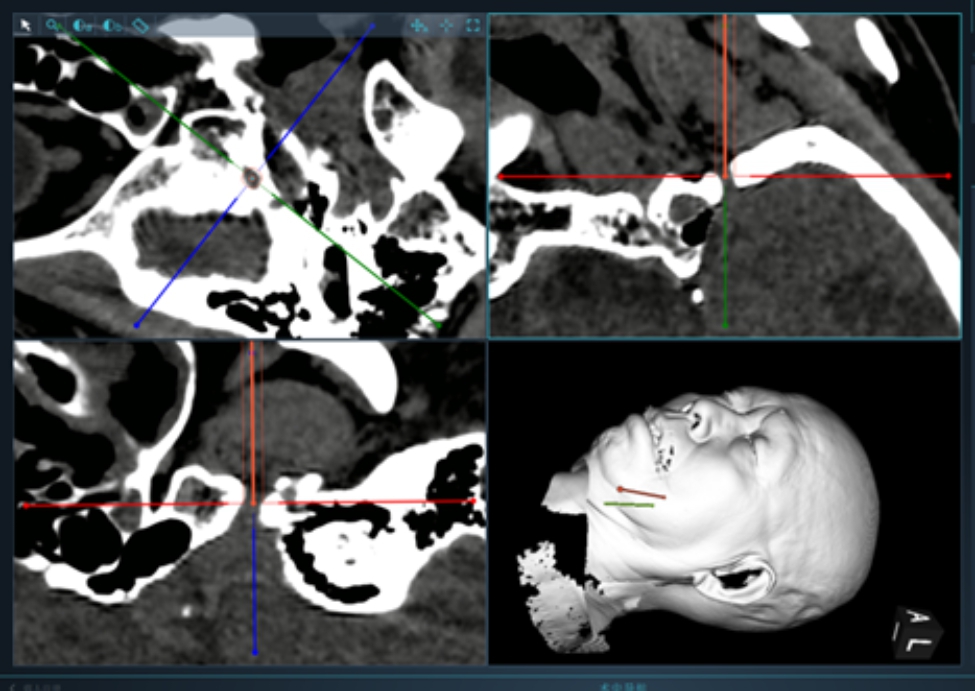
Location of the target and projection of the puncture path on the face

Before the operaions, all patients underwent thin lamina computerized tomography (CT) scan and magnetic resonance imaging test (Siemens, Erlangen, Germany), which mainly included high-resolution T2-weighted SPACE sequences and high-resolution T2-weighted three-dimensional-time of flight magnetic resonance angiography. Preoperatively, CT image data were transmitted to the Sinovation workstation, the line connected the medial third of inner and outer aperture of foramen ovale was used as puncture trajectory (Fig. 3).

### Surgical procedures

**Fig. 4:**
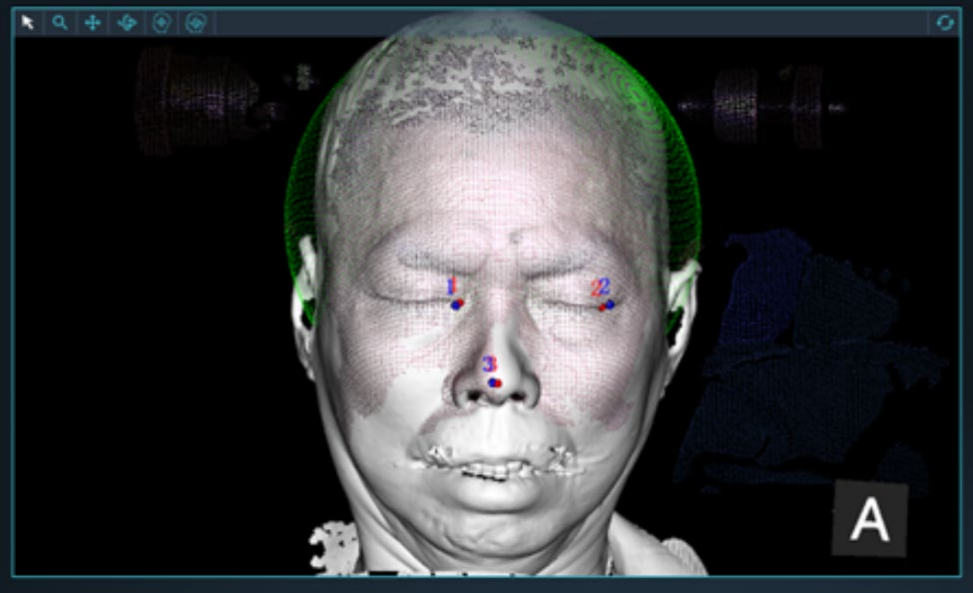
Matching of slected points between the 3D Sstructured light scanning extraction and CT 3D reconstruction

After general anesthesia, all the patients were placed in the supine position with the neck slightly tilted back. The head frame was used for fixing the head and connecting the robot. Surgical plans were uploaded to the robot, then structured light extracted the facial image (Video link: https://cowtransfer.com/s/b8d3c7c9b1a249). The contralateral inner and ipsilateral outer canthus, nasal tip on structured light extraction and CT reconstruction were used for registrations, another point was selected for verification (Fig. 4).

**Fig. 5:**
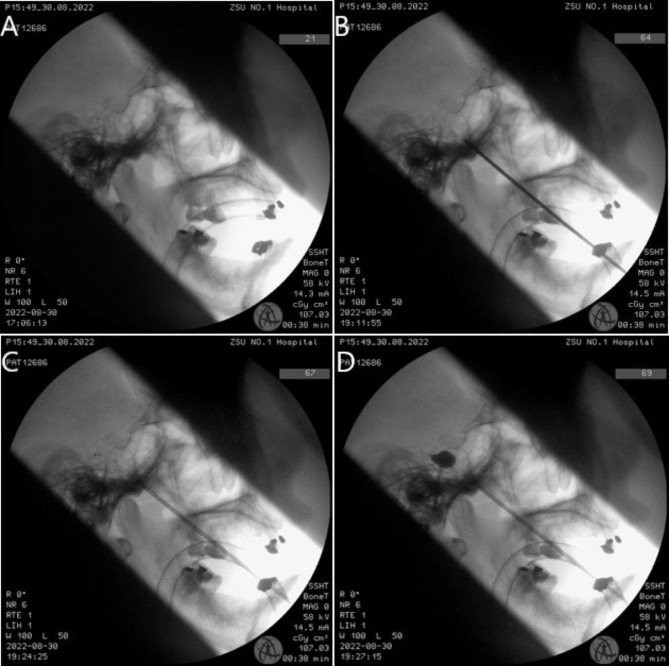
Intraoperative lateral image of the head by C-arm X-ray machine. **(A)** Confirmation of the standard lateral projection angle. **(B)** Puncture of the foramen ovale. **(C)** Insertation of the balloon catheter. **(D)** Contrast agent injection and ganglion compression

After the skin was cut, the needle was advanced to the pre-designed trajectory under the guidance of the robot. The location of the needle was verified by C-arm X-ray machine. After that, we pulled out the needle and imbedded one-time neurosurgical balloon catheter to 5 millimeters behind the clivus line. 0.1 to 1 milliliter ivoersal was inserted to the balloon to obtain pear-shaped balloon, then the trigeminal ganglion was compressed for 150–180 s. After that, the instruments were removed, a sterile patch would be used to the puncture site after compression of the puncture point was performed (Fig. 5).

### Efficacy evaluations

Numerical rating scale (NRS) was used to evaluate the facial pain before PBC, after PBC and follow-up. The Barrow Neurological Institute (BNI) pain and numbness intensity score were used to evaluate the postoperative facial pain and numbness. Volume of ivoresal, compression time, registration error, postoperative complications were recorded.

### Statistical analyses

SPSS 26.0 and R language (version 4.2.1) were used. For measurement data, normality test was performed first, mean ± standard deviation was used to describe the normally distributional measurement data. The overall comparisons of multiple time points were analyzed by one-way repeated measures analysis of variance. The median was used to describe the unnormal data. The ggplot2 package of R language was used for boxplot. P < 0.05 was considered as statistical significance.

## Results

### Clinical data

Nine patients with TN were enrolled, all operations were performed by same surgeon. The basic characteristics of the patients were all listed in Table 1. Of note, six patients underwent MVD or other operations before PBC. One patient suffered from advanced hepatocellular carcinoma with a short life expectancy, one patient suffered from myocardial infarction and took anticoagulants for a long time, the other comorbidities were all listed (Table 1).

**Table 1:** Clinical features of the 9 patients who underwent 3D-structured light registration robot-assisted percutaneous balloon compression

Patients	gender	age (years)	affected side	course (Y)	distribution	comorbidities	previous operation	3D-CISS MR findings
1	female	53	right	>10	V2, V3	diabetes	MVD	Teflon adhesion
2	female	67	left	2	V2, V3	hypertension, cerebral infarction	MVD	Teflon adhesion
3	male	58	right	2	V3	hypertension, myocardial infarction	MVD	Teflon adhesion
4	male	72	right	3	V3	hypertension	trigeminal avulsion	NVC
5	male	80	left	2	V1, V2	hypertension	trigeminal avulsion	NVC
6	male	62	left	>10	V2,	NO	trigeminal occlusion	NVC
7	female	59	right	2	V1, V2, V3	NO	NO	negative
8	male	73	left	9	V2	hypertension	NO	NVC
9	male	56	right	3	V2, V3	hepatocellular carcinoma	NO	NVC

MVD microvascular decompression; V1 ophthalmic nerve; V2 Maxillary nerve; V3 Mandibular nerve; Y years; NVC neurovascular conflict

### Accuracies

All 9 patients successfully completed registrations with an average error of 0.68 mm. All trajectories were successfully punctured one-time without any obstacles. After injection of 0.35–0.95 milliliter ivoresal. The pear-shaped balloons were obtained in all patients, among them, the pear-shaped balloons in three patients were obtained after mild adjustments (Table 1).

### Effectiveness

**Fig. 6:**
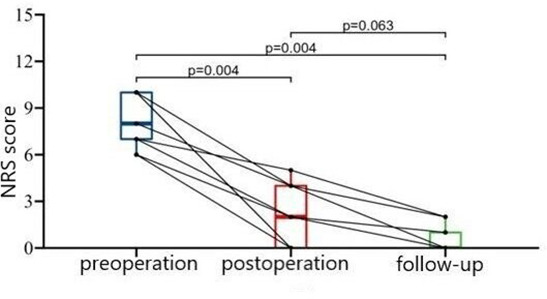
NRS score at different time points

**Fig. 7:**
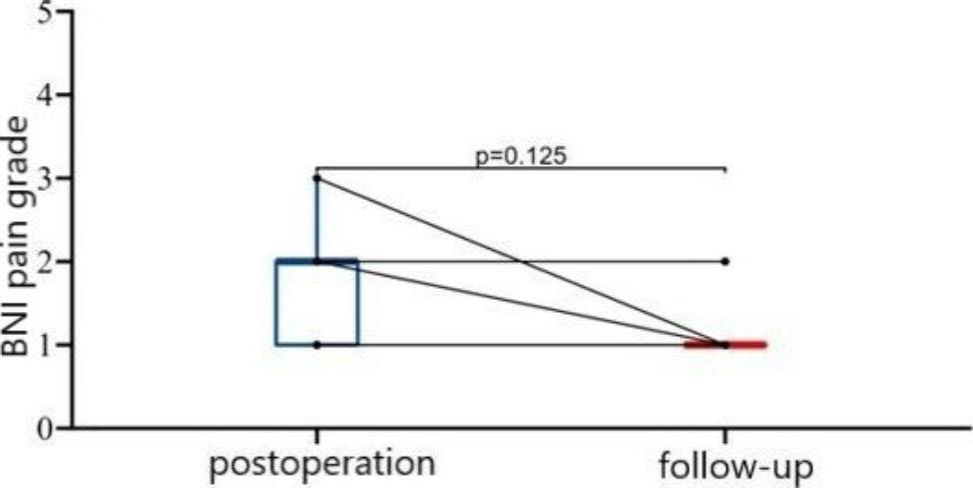
BNI pain intensity score at different time points

The median of NRS scores were 8.0 and 2.0 before and after PBC, which dropped to 0.0 during follow-up. (P < 0.001). It’s showed that the postoperative and follow-up scores were significantly lower than preoperative score (P = 0.004), no significant difference was found between postoperative and follow-up score. (P = 0.063) (Fig. 6) Four patients got immediate pain free (BNI I), five patients got partially pain relief, including three cases with BNI II and two with III (Table 2). The median BNI scores were II after operation and I during follow-up, but no significant difference was found (P = 0.125) (Fig. 7).

**Table 2:** Intraoperative parameters and postoperative outcomes of the patients

Patients	registration error (mm)	volume of ivoersal (mL)	compression durations (s)	pre/postoperative and follow-up NRS score	postoperative and follow-up BNI pain score	postoperative and follow-up BNI numbness score	other complications
1	0.46	0.85	150	10/0/0	1/1	4/2	masseter weakness
2	0.38	0.95	180	7/5/2	3/1	3/2	NO
3	0.79	0.65	180	8/4/0	3/1	3/2	masseter weakness/ herpes labialis
4	0.44	0.9	200	6/0/0	1/1	3/2	masseter weakness
5	1.29	0.35	180	7/2/0	2/1	3/1	NO
6	0.67	0.88	180	6/2/1	2/1	3/2	NO
7	0.86	0.75	180	10/0/0	1/1	2/2	NO
8	0.76	0.4	200	10/4/2	2/2	2/2	NO
9	0.49	0.35	200	10/0/0	1/1	2/2	masseter weakness

BNI Barrow Neurological Institute; NRS numerical rating scale; s second; ml milliliter

### Complications

**Fig. 8:**
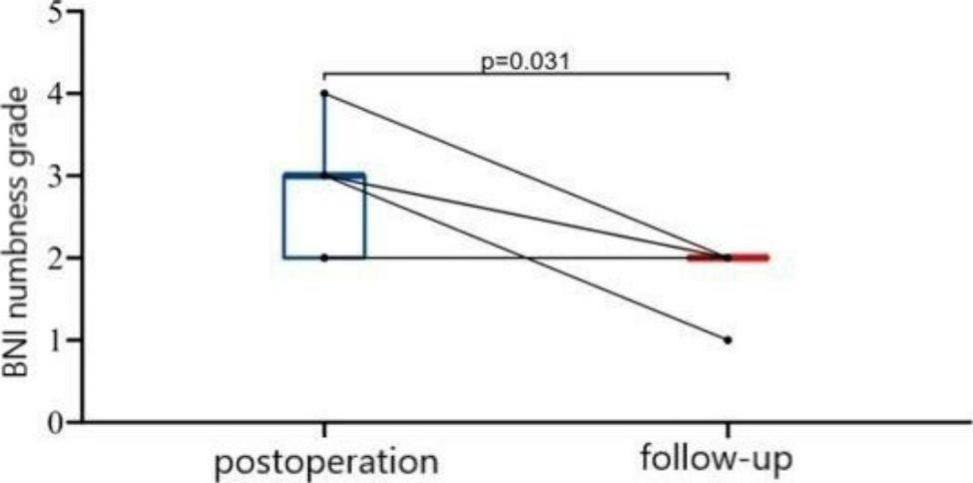
BNI facial numbness score at different time points

All the patients complained of facial numbness, and the BNI scores were II in three cases, III in five cases, and IV in one case, but the symptoms alleviated with time. The median numbness score was III after operation and II during follow-up. (P = 0.031) (Fig. 8) Four patients suffered from transient masseter weakness. No nerve or vascular injury, oral hematoma, keratitis, or infection were found (Table 2).

## Discussions

Oral medication is now the first-line treatment of TN, [1] PBC is also an effective choice [1]. PBC was first introduced by Mullan, [3] puncture foramen ovale, imbed catheter and compression are the three key steps of PBC, puncture foramen ovale and imbed catheter are more vital. We used robot to assist the puncture and C-arm X-ray to verify the locations, the foramen ovale were reconstructed to observe morphologies and design the best trajectories. Of note, the plug can be used for necessary adjustments to verify the punctures. However, the successful puncture of the foramen ovale means nothing to the the successful insertion of the balloon, it is just the first step of PBC. Meckel’s cave doesn’t locate on the trajectory, but locates above the foramen ovale, [9, 10] robotic assistance could greatly convenience this procedure.

### Advantages of robot-assisted foramen ovale puncture

Intraoperative CT can directly verify the accuracy of puncture [8]. In our study, only C-arm fluoroscopy was used to verify the locations of the needle and catheter. What’s more, manipulations of the rigid structures are the main cause of complications, incorrect puncture might cause serve complications, such as carotid-cavernous fistula, subarachnoid hemorrhage, and blindness, [3, 4, 11] but no serious complications were found in our study. Needle and guidewire are rigid structures, while balloon catheter is soft structure. The manipulations of the rigid structures may damage internal carotid artery, brain stem and its vessels, [12] especially in patients with anatomic variation. Robot-assisted PBC could shorten the learning curve, reduce complications and facilitate the promotion of PBC [13]. Such operation could also avoid improper puncture and minimize the risk of complications, reduce radiation, soft tissue damage and scar formation, [13] it could create a favorable condition for repeat operations. In our study, all the punctures were completed one time, the use of robot could improve the accuracy, reduce operation error.

### Advantages of structured light registration

The registration methods of robot-assisted PBC mainly include bone nail registration, sticker registration and structured light registration. Structured light registration markers, such as inner canthus, lateral canthus and nasal tip, are fixed in positions. Although the error of structured light is a little big, [14] we know the error though verifications and make adjustments accordingly. In our study, structured light registration was used and the mean registration error was just 0.68 mm, and all cases were punctured into the foramen ovale one time. What’s more, structure light registration has many advantages, such as contactless, rapid automatic registration and exact verification, CT test on the day of operation and manual adjustments of the markers aren’t required [14]. The patient’s skull can be scanned by point clouds in a wide range, multi-angle and multi-posture, without the restriction from the position of the patients [15] (Video link: https://cowtransfer.com/s/b8d3c7c9b1a249) Therefore, it has significant advantages over other methods.

### Relationships between the shape of the balloon and effectiveness

Many studies highlighted the important role of pear-shaped balloon. The dura forms a “three-fingered glove” around the trigeminal ganglion. As the balloon located in Meckel’s cave, the pressure in the balloon is appropriate to form a pear-shaped head and protrude to the porus trigeminus, and the catheter enter in the Meckel’s cave is the precondition of pear-shaped balloon. The Meckel’s cave is flat before it is filled with balloon, pear-shaped balloon indicates enough pressure in the balloon so the head forms a small protrusion protruding from the porus trigeminus [16]. In addition to typical pear shape, the balloon may also form pear-like, oval, round, irregular, dumbbell shapes [11, 17, 18]. In our study, all the balloons formed pear shape with or without position adjustments of the catheter.

### Trajectories of foreman ovale puncture

Studies introduced various methods to improve the success rates and efficiencies of foramen ovale puncture. C-arm fluoroscopy is now the most widely used, but it only provides two-dimensional planar images, it is difficult to observe foramen ovale, especially in patients with skull base variations [19–21]. Some studies had proposed the use of digital substraction angiography or Dyna-CT to guide this procedure, [22, 23] such methods improved the accuracy of puncture but still require adjustments. Neuronavigation was also used to improve puncture accuracy, but it required real-time monitoring, longer registration time. Personalized three-dimensional printed guide plate was an effective tool for foramen ovale puncture, but the puncture angle and depth of the needle required manual adjustments and controls.

Foramen ovale puncture mainly includes Hartel anterior approach, anterior lateral approach and lateral approach [23] At present, the first and second approaches are commonly used for balloon compressions, and the third approach is mainly used for radiofrequency ablations [23]. Hartel anterior approach and anterior lateral approach take the use of reference point, which depends on the individual. There are differences in the patient’s head and crack mouth size, height of the angle of the mouth varies from one to another, it doesn’t take into account the facial anatomical variations and other conditions. This puncture aims to get the needle into the foramen ovale and mainly used for radiofrequency thermocoagulation, so it might not be the best trajectory for PBC [24].

**Fig. 9:**
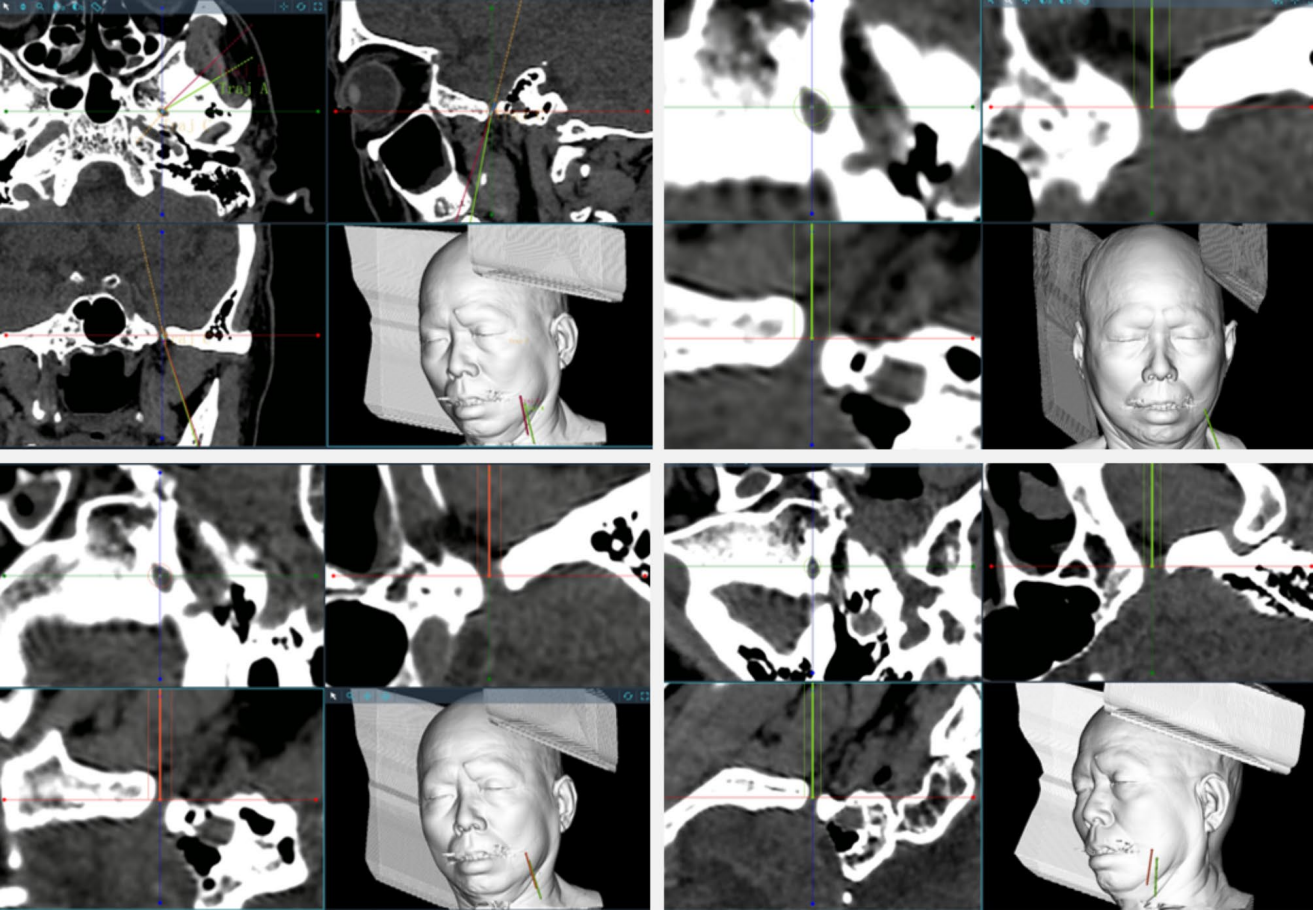
Two trajectories of the foramen ovale, the red line shows the classical Hartel approach. We can find that the classical approach might not be the best in some cases, the trajectory should be designed according to the actual condition, and the puncture point and trajectory (green line) should be adjusted accordingly

What is an ideal puncture trajectory? As we aim to puncture into Meckel’s cave rather than foramen ovale, the path that facilitate the Meckel’s cave puncture is an ideal trajectory. (Fig. 9) One-time successful puncture and adjustments frequency are the two principles of trajectory designations. After the catheter is inserted into foramen ovale, the complex structures in the cave could impede the catheter and result in complications or poor outcomes. In actual condition, foramen ovale is not two-dimension, but a three-dimensional elliptical pipe, robot-assisted operation could design better puncture angle.

To sum up, the advances of artificial intelligence reversed the original thinking of trajectory design. The difference between robot-assisted operationand Hartel anterior method is that Hartel method determines the entry point firstly and then adjusts the puncture angle, while robot-assisted operation determines the target point firstly and then adjusts the path to appropriate angle, no compulsory requirements on the puncture point. Therefore, trajectory could be personalized designed based on the anatomical variations of the foramen ovale and skull base, so the success rate of puncture is greatly increased. It’s like that previous methods use entry point on the sphere to puncture a cylinder inside a sphere, while robot assist surgery in our study selects two points in cylinder to confirm the puncture point on the surface.

## Conclusion

Three-dimensional structured light is a precise registration method for robot-assisted surgery. Robot-assisted PBC is an effective option for patient with TN, the line connects the medial third of the inner and outer aperture is precise and safe trajectory for foramen ovale puncture. More studies are needed for further verifications.

## Data Availability

All data are fully available without restriction by contacting Tao Sun or Chao Yang.

## Abbreviations

TN: trigeminal neuralgia
PBC: percutaneous balloon compression
NRS: Numerical rating scale
BNI: Barrow Neurological Institute
CT: computerized tomography
MVD: microvascular decompression
